# Beyond Systematic and Unsystematic Responding: Latent Class Mixture Models to Characterize Response Patterns in Discounting Research

**DOI:** 10.3389/fnbeh.2022.806944

**Published:** 2022-04-28

**Authors:** Shawn P. Gilroy, Justin C. Strickland, Gideon P. Naudé, Matthew W. Johnson, Michael Amlung, Derek D. Reed

**Affiliations:** ^1^Department of Psychology, Louisiana State University, Baton Rouge, LA, United States; ^2^Behavioral Pharmacology Research Unit, Department of Psychiatry and Behavioral Sciences, Johns Hopkins University, Baltimore, MD, United States; ^3^Department of Applied Behavioral Science, University of Kansas, Lawrence, KS, United States; ^4^Cofrin Logan Center for Addiction Research and Treatment, University of Kansas, Lawrence, KS, United States

**Keywords:** discounting, mixed-effects models, statistical analysis, non-systematic data, latent factor

## Abstract

Operant behavioral economic methods are increasingly used in basic research on the efficacy of reinforcers as well as in large-scale applied research (e.g., evaluation of empirical public policy). Various methods and strategies have been put forward to assist discounting researchers in conducting large-scale research and detecting irregular response patterns. Although rule-based approaches are based on well-established behavioral patterns, these methods for screening discounting data make assumptions about decision-making patterns that may not hold in all cases and across different types of choices. Without methods well-suited to the observed data, valid data could be omitted or invalid data could be included in study analyses, which subsequently affects study power, the precision of estimates, and the generality of effects. This review and demonstration explore existing approaches for characterizing discounting and presents a novel, data-driven approach based on Latent Class Analysis. This approach (Latent Class Mixed Modeling) characterizes longitudinal patterns of choice into classes, the goal of which is to classify groups of responders that differ characteristically from the overall sample of discounters. In the absence of responders whose behavior is characteristically distinct from the greater sample, modern approaches such as mixed-effects models are robust to less-systematic data series. This approach is discussed, demonstrated with a publicly available dataset, and reviewed as a potential supplement to existing methods for inspecting and screening discounting data.

## Introduction

Delay discounting and probability discounting are two key behavioral mechanisms. These are defined as the devaluation of a relevant consequence resulting from the delay or uncertainty associated with its receipt. Clinical research has focused on discounting given empirical work describing and conceptual frameworks positing that many of the behaviors observed in neuropsychiatric (and other) health conditions mechanistically relate to one’s sensitivity to delay and/or uncertainty (Bickel et al., 2014, 2017; MacKillop, 2016; Amlung et al., 2019). For example, research in addiction science has shown that people with substance use disorders have a greater tendency to devalue delayed rewards than healthy controls, a mechanism thought to underlie decisions to use drugs (e.g., cigarettes) and forgo long-term health benefits (e.g., increased later lung cancer risk; MacKillop et al., 2011; Amlung et al., 2017). Although research on probability discounting is more mixed in its relationship with substance use outcomes, similar associations with clinically relevant behaviors have been observed, particularly when outcome-specific discounting tasks rather than monetary-based discounting tasks are used (e.g., probabilistic risk of STI transmission; Johnson et al., 2020). More recently, discounting research has been extended to behavioral addictions such as Internet gaming and gambling, finding comparable predictive associations between specific discounting profiles and health behavior engagement (e.g., Petry and Madden, 2010; Kyonka and Schutte, 2018; Chung et al., 2021). Continued advances in the analysis of discounting data are needed to ensure that the growing emphasis on discounting as a candidate marker of neuropsychiatric health is accompanied by a retained focus on the rigor of the analytic procedures used to generate those conclusions.

The available literature shows that delay and uncertainty tend to decrease reinforcing value and behavioral scientists often regard any deviations from a monotonically decreasing function as erred responding by the participant and/or methodological flaws of the task (Smith et al., 2018). Thus, researchers typically label such deviations as “non-systematic” response patterns. In the seminal account of this issue, Johnson and Bickel (2008) proposed a general framework for assessing whether discounting data are systematic. Previous methods had often used an arbitrary *R*^2^ value when fitting the data to a model such as the hyperbolic decay equation, a method that conflates model fit with the extent of discounting itself as shown by Johnson and Bickel (2008). Rather, they recommended the use of simple rules for the empirical data (i.e., indifference points) based on the most basic expectations of the data. For the data sets they presented, they classified data as non-systematic using two criteria: when (1) an indifference point is greater than the preceding indifference point by a magnitude of 20% of the undiscounted reward value (i.e., JB1), and/or when (2) the last indifference point (i.e., at the largest delay or odds against receipt) is not less than the first indifference point (i.e., shortest delay or smallest odds against receipt) by at least a magnitude of 10% of the undiscounted reward (i.e., JB2). They argued that depending on the data set, the specific criteria should be modified (e.g., adjustment of parameters, dropping the second criterion). They also noted that while the framework can be used to eliminate flagged data, it can be used simply to characterize data without elimination, and if used for elimination, variations such as allowing a single violation of the first criterion may be appropriate. Despite encouragement for such flexibility and some examples of that flexibility (e.g., Johnson and Bruner, 2012; Johnson et al., 2015), the specific non-systematic criteria noted in Johnson and Bickel (2008) have since become the *de facto* gold standard metrics of data quality in the discounting literature. That is, information on systematic responding may be grounds for manuscript rejection or at least substantial revision (e.g., requested to exclude those participants from analysis).

A recent meta-analysis of non-systematic responding in discounting studies sought to identify the prevalence of these patterns in published works (Smith et al., 2018). In their meta-analysis, Smith and colleagues identified 114 discounting experiments in human participants that explicitly reference the use of the Johnson and Bickel (2008) algorithm. Of these, 95 experiments used both criteria from the algorithm (i.e., JB1, JB2), and 14 of those 95 modified the criteria to account for procedural nuances. Across all experiments reviewed, approximately 18% of participant datasets failed at least 1 of the criteria. Rates of non-systematic responding were not found to differ between types of discounting, adults vs. youth, specified samples vs. general samples, hypothetical vs. real/potentially real outcomes, or whether the algorithm was modified. However, non-systematic rates were higher for non-monetary outcomes than monetary ones, as well as higher for university samples versus non-university samples. Findings from Smith et al. (2018) indicated that discounting data are robust and reliable concerning systematic patterns; however, the finding that 18% of datasets featured some degree of non-systematic responding is concerning and questions remain regarding the factors that account for these deviations.

### Latent Class Analyses, Mixed Models, and Discounting Data

As an alternative to set criteria for characterizing discounters (i.e., systematic, non-systematic), Latent Class Analyses (LCAs) can be performed to explore subgroups of responders that comprise a given data set (e.g., systematic, mostly systematic, non-systematic, and so on). The term LCA refers to a collection of methods that are used to extract classes from data (Hagenaars and McCutcheon, 2002; Muthén, 2004). Class membership here refers to a latent feature, extracted from variance in the data, that distinguishes groups or classes of individuals that appear to be distinct from others within the overall sample (Lazarsfeld and Henry, 1968; Weller et al., 2020). Broadly, LCA and derivatives of this methodology are often used as a way of characterizing latent groups concerning some phenomena (Muthén, 2004; Proust-Lima et al., 2015). Derivatives of LCA expand upon the general process, which includes categorical variables, to evaluate changes in class membership over time (Latent Transition Analysis), to evaluate differential shapes and patterns of growth [Latent Class Growth Analysis (LCGA)], and to determine class membership while simultaneously modeling individual-level changes [LCGA + Mixing Modeling (LCMM), Muthén and Muthén, 2000]. Before discussing LCMMs further, we note that LCA is distinct from other clustering approaches (e.g., K-means), wherein the emphasis is on minimizing the distance between some metric (e.g., rate parameter *k*) and the values associated with each of the *n* fitted clusters. In data-driven approaches such as K-means, classes are determined by a process of minimizing individual data distance from *n* centroids, and class membership is established based on proximity to the nearest centroid. That is, such approaches view class membership as determined by proximity rather than probability. Approaches such as K-means are readily applied to large datasets and demonstrate reliable convergence; however, such approaches are more strongly influenced by initial starting values, outliers, and conditions where cluster sizes vary significantly in terms of density and size (Morissette and Chartier, 2013).

Derivatives of the LCA such as LCMM can be extended to include linear modeling and to accommodate a range of longitudinal data types (e.g., continuous, binary; Proust-Lima et al., 2015). The flexibility provided by LCMM is particularly suited to evaluating patterns of choice over time, such as discounting phenomena. When used in this context, LCMMs can be applied to patterns of intertemporal choice over time to identify sub-classes of decision-makers that comprise the greater sample. This approach is distinct from approaches such as K-means because class membership is based on modeling differences (e.g., slopes) across individual data rather than data distance from centroids. Furthermore, class membership in LCMMs is probabilistic for individuals and this differs from approaches such as K-means. For example, a sample is likely to be comprised of multiple classes (with larger samples likely manifesting greater classes) and the results of LCMM explore class membership in a probabilistic sense. That is, the variance regarding individual choice over time is analyzed and viewed in terms of the classes in which it most probabilistically emerged from. This is key in viewing the distinguishing between LCMM and K-means; that is, latent features are extracted from the results of a model and the results probabilistically determine which class best characterizes the individual’s responses. In a relevant example of this approach, Campbell et al. (2021) applied a derivative of LCA – Latent Profile Analysis (LPA) – to evaluate various continuous outcomes (e.g., discounting rate, indicators of demand). Using a latent approach with continuous indicators, the authors found three distinct classes of college students who engage in heavy drinking: low reward value, high discounting (LRHD); moderate reward value, low discounting (MRLD); high reward value, high discounting (HRHD). These profiles corresponded with individuals demonstrating a low demand for alcohol but high rates of discounting, a medium level of demand for alcohol, but low rates of discounting, and high levels of demand and discounting, respectively.

Although the Campbell et al. (2021) study provides an excellent exemplar of methods derived from LCA to indicators of demand and decision-making *across* various tasks, the goal of the current work is more general and specific to responding *within* a decision-making task. That is, the sample of decision-makers in a discounting task is likely to include classes of responders that demonstrated monotonically decreasing choices (i.e., systematic) and those who varied from that expected trend (i.e., non-systematic). These non-systematic responders are likely to demonstrate characteristically different patterns of choice as *compared to* the overall sample (e.g., ascending trends in the presence of increasing delays). In this way, LCMM provides a means to detect responders that behave uncharacteristically of the greater sample and this provides information that may be useful to researchers when deciding how to analyze responding in these tasks.

Despite recommendations by Johnson and Bickel (2008) to adapt a flexible framework, and examples of the adaptive use of the proposed framework (e.g., Johnson and Bruner, 2012; Johnson et al., 2015), many researchers continue to use these criteria rotely. That is, the criteria are being used to distinguish between orderly decreasing data and data that does not conform to this pattern. We propose the use of LCMMs as an alternative to assuming that a single “true” pattern of discounting exists (i.e., systematic vs. unsystematic). That is, LCMMs can be applied to the data to characterize the various subgroups that behave in characteristic and uncharacteristic ways (e.g., increasing value with delays).

### Research Aims

The goal of this study was to test the use of LCMMs with a publicly available data set. The Human Connectome Project (HCP) was a large-scale open-science collaboration sponsored by the National Institutes of Health. The HCP provides a repository of delay discounting data drawn from healthy young adults participating in neural and behavioral research (full recruitment and screening procedures are found in Van Essen et al., 2013). Included among the battery of assessments were two adjusting-amount tasks (Du et al., 2002) that measured delay discounting across $200 and $40,000 reward magnitudes. Previous research has found that HCP delay discounting data are well characterized by hyperbolic-like discount functions (Yeh et al., 2021), exhibit the reward magnitude effect (Naudé et al., 2021; Yeh et al., 2021), and demonstrate the well-published association between cigarette smoking and greater discounting (Naudé et al., 2021). The goals of this report were to apply both the two original Johnson and Bickel (2008) criteria and the LCMM approach to evaluate the correspondence between the two different approaches. Specifically, the goal was to evaluate how two different approaches correlated when LCMMs identified clusters of responders that responded in characteristically different ways from the overall sample.

## Materials and Methods

### Participants

A total of 1206 adults were included in the HCP and discounting data was available for 1198 of those participants. As part of an effort to better understand the relationship between neurology and behavior, participants across various ages and demographics completed a range of neuropsychological and decision-making measures. The sample included comparable groups of male (*n* = 550; 45%) and female (*n* = 656; 54%) participants. The amount of participants in each of the 22–25, 26–30, 31–35, and 36 + age ranges was 247 (20%), 527 (43%), 418 (34%), and 14 (1%), respectively. Participants in the HCP project completed two hypothetical delay discounting tasks as part of the overall battery of assessments. All data used in this study were drawn from the unrestricted set of data and no demographic information is analyzed here.

### Hypothetical Delay Discounting Tasks

The core battery of the HCP included two discounting tasks. One featured a low magnitude Larger Later Reward (LLR; $200) and another high magnitude LLR ($40,000). Across both amounts, indifference points were calculated across delays of 1 month, 6 months, 1 year, 3 years, 5 years, and 10 years. Indifference points across each delay were calculated using methods consistent with Du et al. (2002). That is, the initial value of the Smaller Sooner Reward (SSR) at each delay was 50% of the LLR and the value of the SSR was adjusted following participant choices. Specifically, the value of the SSR would increase and decrease following the choice to select the LLR and SSR, respectively. The degree of adjustment for the SSR was half of the starting SSR value and halved in each subsequent iteration. Following a total of five choices, the final SSR value was considered the indifference point for that delay. This process was repeated for each delay, in ascending order, across both tasks. The results of each were used to construct a ratio of area under the interpolated series to the total area possible, i.e., point-based area under the curve (AUC; Myerson et al., 2001). In the presence of a monotonically decreasing data series, AUC provides a summary index of individual discounting (see Gilroy and Hantula, 2018, for a discussion on AUC interpretation).

### Analytical Strategy

Participant responses on each of the discounting tasks included in the HCP were analyzed using multiple methods for characterizing discounters. Specifically, the criteria in Johnson and Bickel (2008) were compared to the best-fitting LCMMs for each of the Hypothetical Money Choice Tasks (HMCTs). These two approaches are expected to correspond to an unknown degree, with the Johnson and Bickel (2008) approach reflecting comparison to an absolute standard (i.e., JB1, JB2) and LCMMs relative to the trends observed in the sample overall. The methods used to apply the Johnson and Bickel (2008) indicators were adapted from source code included in the *discountingtools* R package (Gilroy, 2017). LCMMs were applied to the HCP data set using the *lcmm* R package (Proust-Lima et al., 2015) and the R Statistical Program (R Core Team, 2021). Data from each of the HMCTs were supplied to the *lcmm* method included in the R package. The *lcmm* package provides considerable flexibility in specifying models; however, this exploration used the most basic linear model to characterize individual data across delays. The use of a basic linear model was selected because it presented the simplest option to index the direction and rate of change for individual choices over time. Indeed, there are various competing options for representing the shape of individual discounting processes (e.g., exponential, hyperbolic) and the use of the linear model provided the simplest model with which to perform LCMM. Furthermore, in regards to comparison with the Johnson and Bickel (2008) comparison, we note that the rules provided did not reference any specific shape for the discounting process.

The *lcmm* method was used to evaluate the overall sample with *n* latent classes and these various fits were evaluated using the Sample-size Adjusted Bayesian Information Criterion (SABIC; Lubke and Neale, 2006). Briefly, the SABIC is a derivative of the Bayesian Information Criterion (BIC; Schwarz, 1978) adjusted for sample size. Lubke and Neale (2006) conducted various simulation studies and their results suggested that the Akaike Information Criterion (AIC; Akaike, 1974) and SABIC fared better overall as indices for determining mixture model fitness. The grouping structure with the lowest SABIC was inspected to evaluate subgroups of discounters.

## Results

### Empirical Evaluations of Discounting Data

The results of screening using the Johnson and Bickel (2008) criteria are displayed across both decision-making tasks in Table 1. Results from the $200 task indicated that 80, 93, and 76% of the sample satisfy (i.e., were not flagged as non-systematic with) JB1, JB2, and both criteria, respectively. Similarly, results from the $40,000 task indicated that 81, 87, and 71% of the sample satisfied JB1, JB2, and both criteria, respectively. Across the sample, 59% (*n* = 715) demonstrated patterns of responding that satisfied both JB1 and JB2 across both tasks.

**TABLE 1:** Johnson and Bickel criteria applied to discounting tasks overall.

$200 Decision-making task		
	Count	Percentage
Systematic Local (JB1)	970	80.96
Systematic Global (JB2)	1125	93.91
Both Systematic	920	76.69

**$40,000 Decision-making task**		

	**Count**	**Percentage**

Systematic Local (JB1)	978	81.64
Systematic Global (JB2)	1045	87.23
Both Systematic	860	71.79

### Class-Based Characterization of $200 Discounting Task

**FIGURE 1:**
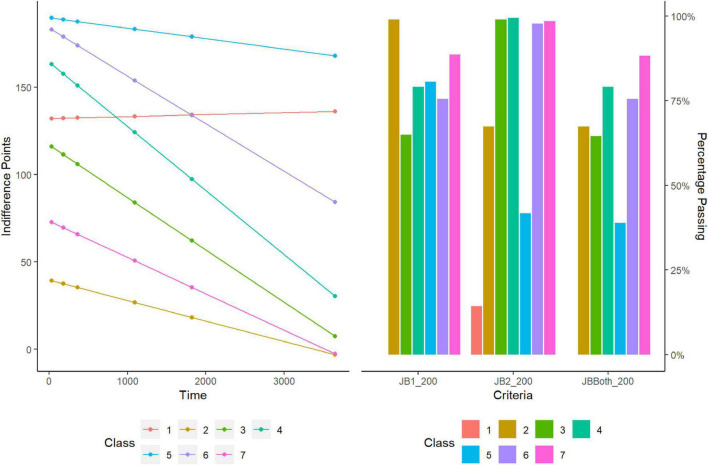
Latent class analysis and systematic criteria of low magnitude task ($200).

**FIGURE 2:**
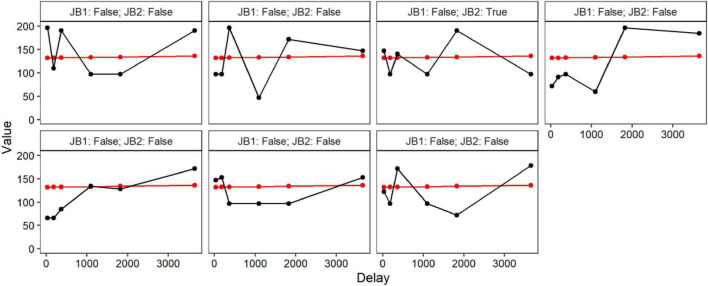
Composition of Non-systematic discounter class.

LCMMs were performed for the $200 discounting task and model fitness across each solution is displayed in the upper portion of Table 2. Model comparisons using SABIC favored a seven-class solution. A visualization of the favored solution is illustrated in the left panel of Figure 1. The range of decision-making patterns in the $200 dataset appeared best characterized by the presence of seven distinct subgroups of discounters. Among these subgroups, six demonstrated patterns of discounting that varied in terms of the estimated intercepts and rates of discounting. Additionally, modeling revealed that one subgroup did not correspond with most decision-makers in the sample. Whereas most demonstrated a trend of decreasing value as a function of time, this subgroup demonstrated responding in the opposing direction, see Figure 2. Additionally, 6 of the 7 responders failed both of the Johnson and Bickel (2008) criteria and 1 of the 7 failed the first of the Johnson and Bickel (2008) criteria. Additional information regarding the rates of systematic responding is provided in the right panel of Figure 1 and Table 3. Information regarding the distribution of AUC within the $200 task across each of the classes is provided in Table 4.

**TABLE 2:** Evaluation of latest classes across discounting tasks.

	Fits with *N* Classes (200 USD)							
	1	2	3	4	5	6	7	8
SABIC	75570.0	75581.7	75144.0	75131.6	75126.0	75152.0	**75122.9**	75134.1
AIC	75558.5	75564.5	75121.1	75102.9	75091.6	75111.8	**75077.0**	75082.4
Class 1%	100	∼100	23	3	<1	25	<1	0.668
Class 2%		<1	5	22	49	4	8	7
Class 3%			70	7	30	15	24	47
Class 4%				65	4	6	14	14
Class 5%					16	44	3	24
Class 6%						3	3	3
Class 7%							44	3
Class 8%								<1

	**Fits with *N* Classes (40,000 USD)**							
	**1**	**2**	**3**	**4**	**5**	**6**	**7**	**8**

SABIC	75897.2	75623.2	75265.2	75115.4	75043.5	**75039.4**	75054.2	
AIC	75885.7	75606.0	75242.2	75086.8	75009.1	**74999.3**	75008.3	
Class 1%	100	36	36	22	26	24	26	
Class 2%		63	43	34	24	23	24	
Class 3%			19	35	22	20	22	
Class 4%				7	19	15	15	
Class 5%					6	11	<1	
Class 6%						4	8	
Class 7%							3	
Class 8%								

*The best-performing model amongst fits is bolded for each dataset.*

**TABLE 3:** Latent class linear mixed modeling across discounting tasks.

		200 USD	
	
Class	% Systematic local (JB1)	% Systematic global (JB2)	% Both systematic
1 (*n* = 7)	0	14	0
2 (*n* = 101)	100	67	67
3 (*n* = 299)	64	98	64
4 (*n* = 172)	79	99	79
5 (*n* = 36)	80	41	38
6 (*n* = 45)	75	97	75
7 (*n* = 538)	88	98	88

		**40,000 USD**	
	
**Class**	**% Systematic local (JB1)**	**% Systematic global (JB2)**	**% Both systematic**

1 (*n* = 295)	82	98	81
2 (*n* = 284)	89	59	54
3 (*n* = 245)	79	97	78
4 (*n* = 180)	75	98	75
5 (*n* = 135)	75	94	75
6 (*n* = 59)	83	61	55

**TABLE 4:** Distribution of point-based area under curve.

	$200 Choice task			$40,000 Choice task		
Class	M (SD)	Mdn (Q1-Q3)	N	M (SD)	Mdn (Q1-Q3)	N
1	0.66 (0.05)	0.62 (0.57–0.68)	7	0.42 (0.09)	0.36 (0.21–0.43)	295
2	0.05 (0.03)	0.03 (0.02–0.04)	101	0.88 (0.07)	0.82 (0.64–0.88)	284
3	0.28 (0.07)	0.22 (0.09–0.28)	299	0.67 (0.08)	0.61 (0.49–0.67)	245
4	0.46 (0.08)	0.41 (0.27–0.47)	172	0.25 (0.09)	0.18 (0.09–0.25)	180
5	0.89 (0.08)	0.84 (0.71–0.9)	36	0.13 (0.07)	0.08 (0.04–0.11)	135
6	0.7 (0.07)	0.65 (0.58–0.69)	45	0.06 (0.05)	0.03 (0.02–0.04)	59
7	0.13 (0.07)	0.08 (0.04–0.13)	538	—	—	—

### Class-Based Characterization of $40,000 Discounting Task

**FIGURE 3:**
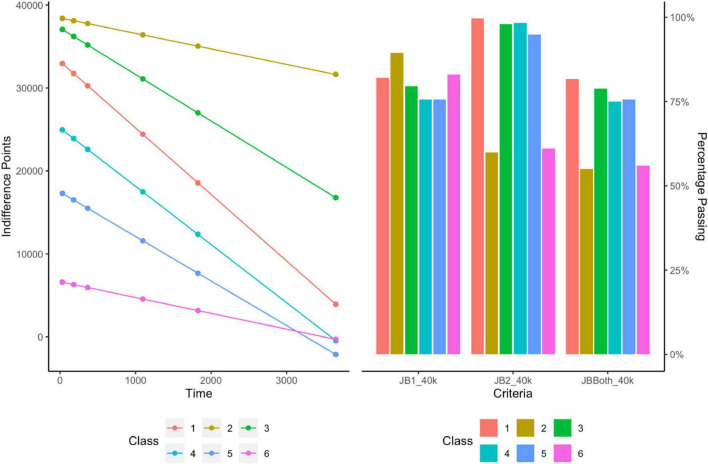
Latent class analysis and systematic criteria of high magnitude task ($40,000).

LCMMs were performed for the $40,000 discounting task and model fitness across each solution is displayed in the lower portion of Table 2. Evaluations of model fitness using SABIC favored the six-group solution. A visualization of the favored solution is illustrated in the left panel of Figure 3. The range of decision-making patterns in the $40,000 dataset appeared best explained by the presence of six distinct subgroups of discounters that varied in terms of intercept and rate of discounting. The analysis did not indicate that there was any particular subgroup that varied meaningfully in terms of trends across increasing delays (i.e., in direction). As expected, the various subgroups of responders passed the Johnson and Bickel (2008) criteria in varying degrees, see the right panel of Figure 3 and Table 3. Details regarding the distribution of AUC values within each class in the $40,000 task are provided in Table 4.

## Discussion

Methods for elucidating and analyzing discounting phenomena continue to be refined, with a growing push toward leveraging more sophisticated methods, such as mixed-effects models (Young, 2017, 2018). Mixed-effects models have many advantages and are more robust to issues that may exist regarding responders at extremes, e.g., non-systematic patterns (Young, 2017). However, it is common and expected for researchers evaluating discounting phenomena to characterize and describe the decision-makers that comprise the full sample. Although an improvement over previous methods based on the *R*^2^ metric (Johnson and Bickel, 2008), based on historically observed behavioral patterns, and easily performed, the framework presented in Johnson and Bickel (2008) is only one means of characterizing a discounting dataset. As such, many approaches likely exist and are associated with benefits and drawbacks.

The approach reviewed in this work (LCMM) provides a novel means of evaluating for the presence of subgroups that appear qualitatively different than others in the sample. This extends the earlier Johnson and Bickel (2008) method by allowing the standards for expected patterns to be derived from the sample itself (i.e., what the data will be compared against), rather than an *a priori* expectation of how individuals should respond in all instances. That is, no presumptions are necessary and researchers need not rely on any general criteria to make analytical decisions. However, it should be noted that the lack of presumptions is not a universal positive. The Johnson and Bickel (2008) framework uses presumptions based on scientific observation to determine if data may be suspect. As such, the features noted in Johnson and Bickel (2008) provide a way to reference the greater population of decision-makers beyond the immediate sample and across multiple samples. The present framework of LCMM, while based on the data itself with no presumptions, categorizes participants into subgroups but is silent on whether responding deviates from expectations of orderly responding overall (i.e., correspond with the greater population from which they are derived). Furthermore, the number of subgroups is also likely to vary considerably across samples—with larger numbers of subsets being more likely with larger datasets.

In discussing how LCMMs can help guide discounting analysis, several points warrant noting as they relate to mixed-effects models. First, mixed-effects modeling already provides some means of accommodating responders at extremes because the manner of optimization (e.g., Maximum Likelihood Estimation) typically pulls estimates toward the group mean (Young, 2017). This effect, shrinkage, has the added benefit of drawing the more extreme (e.g., very low, very high) responses toward the mean of the group. These more extreme responses are typically those that result in participants failing one or more of the criteria provided in Johnson and Bickel (2008). As such, mixed-effects modeling alone can accommodate such challenges, to a degree. Second, it is necessary to note that the mixed-effects approach rests on the assumption that individual fits/estimates emerge from the same distribution of parameter values as the respective group. If the overall sample includes individuals or subgroups that differ characteristically from the overall sample (e.g., increasing rather than decreasing trends), then it is more appropriate to treat and analyze these groups separately (i.e., remove them from the planned analysis). When paired together, the LCMM approach complements the strengths of mixed-effects models quite nicely in this specific regard.

In furthering the argument for both LCMMs and mixed-effects modeling, this evokes questions regarding the framework provided by Johnson and Bickel (2008) and how these conventions fit in a data-driven approach. Indeed, the Johnson and Bickel (2008) criteria have been used as a proxy for data quality and the available literature is largely restricted to data that is considered to be “systematic” in nature. To address some of these questions, we wish to clarify that the Johnson and Bickel (2008) criteria have utility beyond their typical use as the basis for including or excluding responders. For instance, these have good descriptive utility for characterizing responding within a dataset and provide an easily interpreted index with which to appraise an overall sample. Indeed, this provides a standard with which to classify trends in responding that can be compared across various samples. As such, it is reasonable to apply both the Johnson and Bickel (2008) and LCMMs but base decisions on what data are included in mixed-effects modeling based on the clusters identified in the LCMMs. However, it warrants reiterating that using LCMMSs to include or exclude data relies on an individual data set’s classification within the overall data set, meaning that decisions to include or exclude would be based on the entire class rather than on the specifics of any individual data set.

### Limitations

Although data-driven, robust, and applicable to discounting, LCMMs do present several limitations. First, LCMMs entail both considerable flexibility and considerable complexity (van de Schoot et al., 2017). For example, the *lcmm* method applied provides a range of options for the researcher to fit individual data (e.g., linear, n-spline), to compare mixture models (e.g., 1- vs. 2- vs. 3-group fits), and to explore how many clusters might exist (e.g., 2 vs. 10). The decision to use a linear model in this evaluation was effective for providing initial support for LCMMs in this specific regard; however, additional research and study with models more commonly used in the literature is warranted (e.g., n-spline, hyperbolic, hyperboloid). Second, and this challenge is shared with mixed-effects modeling, computational requirements scale poorly with complexity. Even with modern hardware, individual LCMMs may take several minutes, perhaps hours, to converge with complex data sets. Third, few guidelines currently exist with which to perform and then evaluate the relative fitness of LCMMs (Weller et al., 2020). For example, initial fits can be judged based on the AIC, BIC, the SABIC, or the log of the likelihood itself, but ultimately, the analyst has considerable freedom concerning model building (e.g., to vary the number of classes or covariates; van de Schoot et al., 2017). As such, LCMMs entail far more complexity than the algorithm-based approaches to screening discounting data. Fourth, we acknowledge that level of access to HCP demographic data for the present report was restricted to broad sample estimates (e.g., multi-year age range) which precluded a careful analysis of associations between participant characteristics and latent-class membership. We note, though, that HCP data were drawn from healthy young adults with no pre-existing psychiatric or neuropsychiatric disorders, thereby representing only a subset of the larger population and potentially constraining the range of response variability used to identify latent classes. Lastly, the LCMM approach used in this study was evaluated with just one publicly available dataset. As such, researchers should continue to evaluate multiple methods for characterizing individual discounting patterns.

Although LCMMs and mixed-effects modeling increase the complexity of work in discounting, we suggest that researchers in this area consider the use of these methods for several reasons. First, mixed-effects methods already provide a means of accommodating responders at extremes (Young, 2017, 2018). Indeed, these methods should be explored before conducting planned analyses but should not be considered a replacement for carefully inspecting the empirical data. Second, when used together, both empirical reviews and LCMMs can be used to explore the degree to which the data correspond within the sample as well as to the greater population from which they are assumed to emerge. Combining these approaches balances the desire to both retain as much data as possible and exclude data that might limit the generality of the analysis.

## Author’s Note

The source code necessary to recreate this work is publicly hosted in a repository at: https://github.com/miyamot0/LatentClassDiscounting. The terms of the Human Connectome Project require that all parties request access to the data from the following location: https://www.humanconnectome.org.

## Data Availability Statement

Publicly available datasets were analyzed in this study. This data can be found here: http://www.humanconnectomeproject.org/.

## Ethics Statement

The studies involving human participants were reviewed and approved by The Human Connectome Project and was managed and organized by the National Institute of Health. The patients/participants provided their written informed consent to participate in this study.

## Author Contributions

SG, JS, and DR: study conception and design. SG, JS, DR, and MJ: analysis and interpretation of results. SG, GN, JS, DR, and MJ: draft manuscript preparation. All authors reviewed the results and approved the final version of the manuscript.

## Conflict of Interest

The authors declare that the research was conducted in the absence of any commercial or financial relationships that could be construed as a potential conflict of interest.

## Publisher’s Note

All claims expressed in this article are solely those of the authors and do not necessarily represent those of their affiliated organizations, or those of the publisher, the editors and the reviewers. Any product that may be evaluated in this article, or claim that may be made by its manufacturer, is not guaranteed or endorsed by the publisher.
